# The Tuberculin Test of Cattle as a Guarantee of Tubercle Free Milk

**Published:** 1910-11-12

**Authors:** 


### SPECIAL ARTICLE.

### THE TUBERCULIN TEST OF CATTLE AS A GUARANTEE OF TUBERCLE FREE MILK.

That there are two entirely separate types of tuberculosis in human beings is universally admitted—phthisis without or with tuberculous laryngitis, tuberculous ulceration of the bowel, and possibly tuberculosis of the kidney, bladder, or epididymis; and caseous cervical, bronchial, mediastinal or mesenteric glands, spinal caries, lupus vulgaris, scrofulodermia, tuberculous arthritis, especially of hip, knee, ankle, wrist, or elbow, tuberculous dactylitis, tuberculous peritonitis, caseous cerebral nodules, tuberculous meningitis, general tuberculosis. The second main group is sometimes spoken of as surgical tuberculosis; it is practically always due to infection from tuberculous milk.

### Tuberculous Milk.

The difficulty is to know how to make certain that one's milk-supply is free from tubercle bacilli. The percentage of infected samples is so great that unless special precautions are taken it is almost certain that every child in a large town, or even in many country places, drinks tubercle bacilli in its milk every year, if not every month or every week. It is a pity to boil or to pasteurise the milk, for this treatment renders it less palatable. It also destroys some of the nutritive properties; infants thrive less well on boiled than they do on unboiled milk, and the same applies almost as much to pasteurised milk. The difficulty is to know how best to be certain that the milk from a given cow is free from the tubercle danger. Is the absence of reaction when the ordinary tuberculin test is applied to the cow a safe criterion? This has been the subject of some very careful inquiries, and the results are very important in their bearing on infant feeding with cow's milk. Dr. Bolle, Dr. Schlungbaum, and a veterinary surgeon, Dr. Schroeder, carried on extensive investigations in the Bolle dairy, the milk of which is largely used for children. The number of cows under observation between 1907 and 1909 was 304. Each was given injections of tuberculin on admission, and thereafter twice or three times a year. The initial dose was 0.5 c.c., increased later to 1 and even to 2 c.c., in order to induce a more decisive reaction whenever possible. It is noteworthy that even with the larger inoculations the reactions were frequently contradictory. The first criterion of a positive reaction was taken to be the rising of the temperature to 40° C. and over, provided the difference between the temperature before and after injection was 1° C. or more. Later the reaction was considered positive when the difference in temperature was only 0.5° C., provided that the temperature chart showed a febrile curve. The cows were also inspected every two weeks by two veterinary surgeons, and every month by the official veterinary surgeon of the district also. Every day a guinea-pig was injected with milk from the mixed supply from the dairy as a whole; every fortnight a guinea-pig was injected with milk from the mixed produce of each shed of about twenty cows; and three or four times a year the milk of each individual cow was similarly tested separately. Any cow exhibiting the slightest disease of the udder was at once isolated from the rest, and when the tuberculin gave a positive reaction the animal was separated from the herd.

### Conclusions at the Bolle Dairy.

Open tuberculosis was never recognised clinically in the dairy; but seventy-five cows gave a positive and nine a doubtful reaction. Out of these eighty-four only in one case were virulent tubercle bacilli actually demonstrated in the milk by the guinea-pig inoculation test. Moreover, seventeen cows gave both negative and positive reactions at different times, irrespective of the dosage of the tuberculin. The authors of this particular investigation are therefore of opinion that the tuberculin test is too stringent, and that it excludes many cows from the dairy unnecessarily. Unfortunately the results of post-mortem examination in those cows which, having given a positive tuberculin reaction, were fattened and sent to the butcher are not recorded; and although one can feel for the dairyman when he is obliged to send away a good milch cow that looks in perfect health, simply on the ground that it reacts positively to tuberculin, nevertheless, in the interests of the children whom medical men are safeguarding it would certainly seem better to err upon the safe side than to permit milk to be freely supplied from cows that react positively to tuberculin injections. The public as a whole should be willing to pay more for the best mik for the sake of the safety of the infants; if parents demand milk above suspicion the dairies must satisfy every condition including the tuberculin test. That the latter is essential is indicated by the fact that the milk of one of the cows in the Bolle dairy which gave a positive reaction contained virulent tubercle bacilli. Doubtless other cows with virulent bacilli would have been found had not all cows giving a positive reaction been separated from the rest at once, for the reaction reveals early tuberculosis before it has given rise to any general evidence of ill-health. The reliability of the tuberculin test is confirmed on all hands in so far as it indicates tuberculosis somewhere in the cow, though not necessarily tubercle bacilli in its milk. The authorities at Sao Paulo, in Brazil, have published the results of measures adopted by them, according to which all cattle are repeatedly tested with tuberculin, and every beast that reacted positively was destroyed. Many such animals looked perfectly healthy on mere inspection, and the action of the authorities aroused much indignation amongst owners; but in all cases that were investigated after giving a positive tuberculin test tuberculous foci were found, though they might be limited to the mesenteric glands. However useful the clinical examination of cattle may be, it cannot compare in delicacy and certainty with the tuberculin test, especially in cases when the tuberculous lesions are early. Even tuberculosis of the udder may exist for some time without being clinically recognisable; and yet the small active foci in the healthy-looking udder of the healthy-looking cow may cause the milk to be teeming with virulent tubercle bacilli. The guinea-pig method of determining this takes a month or six weeks; in the meantime, if the milk of the tuberculin-reacting cow is included with that of the other cows in the dairy, the whole of the milk-supply becomes dangerous even if the infection does not spread to any of the other cows. The consumer, if he has any doubt whatever, has no alternative but to boil or pasteurise the milk if he wishes to be certain that his child is running no risk of caseous glands or spinal caries. The milk so treated is not so nutritious as is good fresh milk. The subject inevitably comes back to the necessity for periodic testings of dairy cows with tuberculin, and for the eliminating from the herd any beast that gives a positive reaction.

